# Fabrication of Flame-Retardant Ammonium Polyphosphate Modified Phytic Acid-Based Rigid Polyurethane Foam with Enhanced Mechanical Properties

**DOI:** 10.3390/polym16152229

**Published:** 2024-08-05

**Authors:** Xu Zhang, Zhaoqian Wang, Shuai Ding, Zhi Wang, Hua Xie

**Affiliations:** 1 Liaoning Key Laboratory of Aircraft Fire Explosion Control and Reliability Airworthiness Technology, Shenyang Aerospace University, Shenyang 110136, China; 2 School of Safety Engineering, Shenyang Aerospace University, Shenyang 110136, China

**Keywords:** ammonium polyphosphate, rigid polyurethane foam, flame retardancy, smoke toxicity, mechanical properties

## Abstract

Ammonium polyphosphate (APP) and self-made nickel phytate (PANi) were used as modified materials to prepare green biomass rigid polyurethane foam (RPUF). The flame retardancy, thermal stability, smoke toxicity and mechanical properties of the modified RPUF were investigated by limiting oxygen index (LOI), a cone calorimetry (CONE) test, thermogravimetric analysis and a compression test. The results showed that the RPUF with 10 wt% APP (PANi/APP10) had the highest LOI of 26.5%. Its peak heat release rate (PHRR) and total heat release (THR) were reduced by 29.64% and 24.05% compared with PANi/APP0 without APP. And its smoke production rate (SPR) and total smoke release (TSR) decreased by 33.14% and 19.88%, respectively. Compared with pure RPUF, the compressive strength of PANi/APP10 was increased by 50%, mainly because APP itself was an ultra-fine powder, which was better compatible with the matrix and improved the hardness of the material. The results showed that the synergistic effect of the gas phase and the condensed phase mechanism could effectively improve the flame-retardant effect. The current research results provided a new strategy for the preparation of green and low-toxicity RPUF.

## 1. Introduction

In recent years, polyurethane foam (PUF) has become an extremely important material in various fields [1]. Because of its excellent elasticity, flexibility, elongation and compressive strength [2,3,4], it can be used as a cushioning material with excellent performance for packaging high-end precision instruments, valuable instruments, high-grade handicrafts and so on. In addition, rigid PUF (RPUR) has the characteristics of low thermal conductivity [5], which can be used for building exterior wall insulation, pipeline insulation materials, etc., and can also reduce energy consumption. Meanwhile, flexible PUF (FPUF) can be adjusted through the production process to meet the needs of different applications due to its pore size and distribution [6]. Therefore, it is widely used in the automotive industry and furniture industry, such as in the manufacturing of seats, roofs, doors and other components to provide good comfort and cushioning. In conclusion, PUF plays an important role in many fields due to its excellent properties and diverse uses.

While the RPUF has many excellent characteristics, it also has some shortcomings that cannot be ignored. For example, it is extremely flammable, and when burned it emits large amounts of smoke and toxic gases, which limits its usefulness [7]. Therefore, the development of the RPUFs with low toxicity, low smoke and high flame-retardant efficiency is of great significance for their application in aerospace and other fields.

There are many studies that have attempted to reduce the fire hazard of RPUF. Adding flame retardants to RPUF is the most effective way to improve the flame-retardant properties of the RPUF. Flame retardants can be divided into halogen flame retardants and halogen-free flame retardants [8]. Halogenated flame retardants are now strictly regulated by social regulators due to the harm they cause to human health and the ecological environment [9]. In recent decades, many halogen-free flame retardants have been developed to improve the flame-retardant properties of polymer materials [10]. At present, commonly used halogen-free flame retardants are made of non-renewable materials, which make it difficult to meet the current requirements of environmental protection and sustainable development. In today’s depletion of non-renewable energy sources, biomass flame retardants such as lignin [11], protein [12], starch [13] and chitosan [14] have attracted much attention because of their green and environmentally friendly properties [15].

In recent years, researchers have conducted in-depth research on biomass flame-retardant polymers [16]. Yu et al. prepared a biomass intumescent flame-retardant system with good flame-retardant properties by hydrolyzing silk (HS) and phytic acid (PA) PA@HS and mixing it with potato starch (PS) to prepare flame-retardant polyacrylonitrile (PAN) [17]. The authors found that the tensile strength and elongation at break of PAN at PA@HS and 5% PS increased by 18.8% and 86.3%, respectively. The peak heat release rate (PHRR) decreased by 54.8%, and the peak smoke production rate (PSPR) decreased by 79.3%. Wang et al. used chitosan and furfural as raw materials to prepare a fully bio-based carbonizer (CFU), which was compounded with ammonium polyphosphate (APP) into polylactic acid (PLA) to synthesize a novel composite material with good flame-retardant properties [18]. A UL-94 test V-0 rating can be achieved by adding 3.75 wt% CFU and 11.25 wt% APP to the PLA. In addition, in terms of mechanical properties, the elongation at break and impact strength have been improved. Wang et al. prepared reactive bio-based P/N flame retardants (VTP) from vanillin, tetrazolium and phosphanthrene phenanthrene, and introduced them into epoxy resins (EP) for flame retardancy [19]. The test results showed that the EP with 5% VTP could reach the UL-94 test V-0 level, and its limiting oxygen index (LOI) was increased by 30.5%, PHRR was reduced by 36.1%, and total smoke production (TSP) was reduced by 37.9%. Through the above research, it can be concluded that the addition of biomass flame retardant to polymer materials should be used in conjunction with one or several flame retardants to achieve the best flame-retardant effect.

APP has attracted much attention in the field of flame-retardant polymers due to its advantages of low toxicity, low smoke and environmental protection, and can be used as one of the best choices for synergistic flame retardants [20,21,22]. Zhang et al. prepared RPUFs from biomass soybean oil-based polyols and APP [23]. The authors found that after adding 20% APP, the LOI of RPUF (RPUF-S3-20) reached 21.5% and the PHRR decreased by 12.5%. Zhang et al. used APP/cobalt phytate (PA-Co) blending to prepare RPUF/APP/PA-Co [24]. Compared with pure RPUF, the total heat release (THR) of the RPUF was significantly reduced when APP was added at 40 phr and PA-Co was added at 10 phr. In addition, the addition of APP/PA-Co to the RPUF matrix can form a dense carbon layer during combustion, thereby reducing the emission of flammable materials and flue gas in the composite material. Li et al. modified APP with b-cyclodextrin (β-CD) as an intumescent flame retardant with the addition of RPUF [25]. The experimental results indicated that the PHRR of the sample with 25 wt% β-APP decreased by 43.8% compared with pure RPUF. Liao et al. synthesized a novel intumescent flame retardant (TPAPP) for flame-retardant PLA using tannins (TA), polyethylenimine (PEI) and APP as raw materials [26]. The study found that when 5% TPAPP was added, the LOI of PLA composites increased to 27.0%, reaching the V-0 rating of the UL-94 test. Compared to pure PLA samples, its THR and TSP were reduced by 17% and 77%, respectively. The results showed that TPAPP had a significant synergistic effect on the flame-retardant properties of PLA.

With the increasing scarcity of non-renewable resources, biomass flame retardants are widely developed. This can improve the flame-retardant performance of RPUFs while reducing dependence on non-renewable resources, which is in line with the concept of sustainable development. At the same time, intumescent flame retardants can be used to better improve the flame-retardant performance of the RPUF. Among many biomass flame retardants, phytic acid is rich in P elements, which will produce P· when combusted, capture the H· and HO· around the gas and play an important role in the gas phase flame-retardant mechanism. In addition, PA is derived from plant rhizomes and is an abundant resource, which can effectively alleviate the shortage of non-renewable energy sources [27]. At the same time, PA has a good chelation effect with some metal ions and can prepare green and non-toxic phytate flame retardants. This kind of flame retardant produces less smoke when burning, which not only improves the flame-retardant effect but also reduces pollution to the environment. Therefore, PA flame-retardant polymers have attracted the attention of researchers.

In previous studies, nickel phytate flame-retardant polyurethane foam has been reported less often. In order to better improve the flame-retardant effect, APP is selected as a synergistic flame retardant. This not only introduces P and N elements into the vapor phase flame-retardant mechanism but can also form a dense carbon layer as a carbon source to prevent the release of smoke, as the gas generated by combustion inhibits combustion. Therefore, in the current study, PA and nickel acetate tetrahydrate (Ni(Ac)_2_·4H_2_O) were used as raw materials to prepare biomass flame-retardant PANi by green- and simple-chemical methods. And the low-carbon and environmentally friendly RPUF was prepared by blending APP with PANi as an additive flame retardant in different proportions, and the optimal additional amount of APP was explored. The flame-retardant behavior, thermal stability, smoke toxicity and mechanical properties of the modified RPUF were systematically investigated. In addition, the morphology of carbon residue in the RPUF was studied by scanning electron microscopy (SEM), and the flame-retardant mechanism of PANi and APP in the RPUF was proposed.

## 2. Experiments

### 2.1. Materials

Polyether polyol (3630), triethanolamine (A33), silicone oil (L-580) and dibutyltin dilaurate (DBTDL) were purchased from Zhuolian Zhichuang Polymer Co., Ltd. (Changzhou, China), polyisocyanate methylene (PM200) was provided by Yantai Wanhua Polyurethane Co., Ltd. (Yantai, China), ammonium polyphosphate (APP) was purchased from Shandong Yousuo Chemical Technology Co., Ltd. (Linyi, China), phytic acid (PA, ≥70%) and 95% absolute ethanol was provided by Sinopharm Chemical Co., Ltd. (Shanghai, China), acetic acid tetrahydrate (Ni(Ac)_2_·4H_2_O, analytically pure) was provided by Tianjin Huasheng Chemical Reagent Co., Ltd. (Tianjin, China) and deionized water was prepared by this laboratory.

### 2.2. Preparation of RPUFs

**Figure 1:**
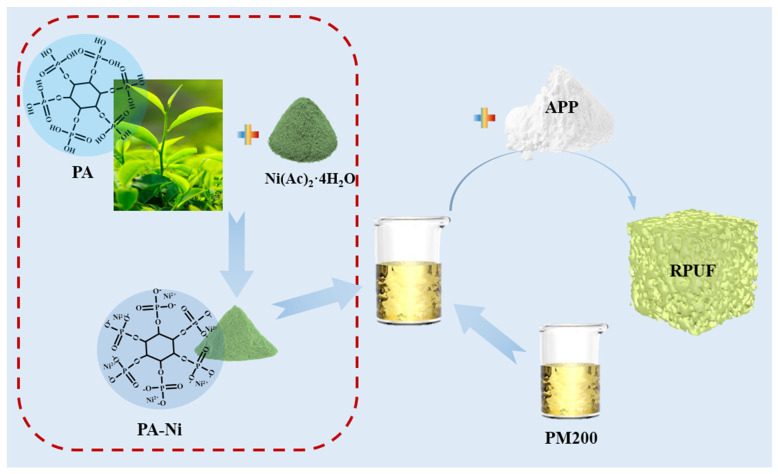
The preparation process of the modified RPUF.

The formulation shown in Table 1 was used for the preparation of RPUF. The preparation process of modified RPUF was shown in Figure 1. First, 3630 polyols, A33, L-580, DBTDL, deionized water, PANi (lab-made) and APP were added to the paper cup in sequence and stirred well with a mechanical stirrer. Then the PM200 solution in each proportion was quickly added and stirred for 45 s, quickly injected into the mold and foamed freely at room temperature for 24 h. Based on previous research, all five samples were supplemented with 3 wt% of PANi [28]. The modified RPUFs with APP content of 2.5, 5, 7.5 and 10 wt% were named PANi/APP2.5, PANi/APP5, PANi/APP7.5 and PANi/APP10, respectively. PANi/APP0 was the control group.

### 2.3. Characterization

**Figure 2:**
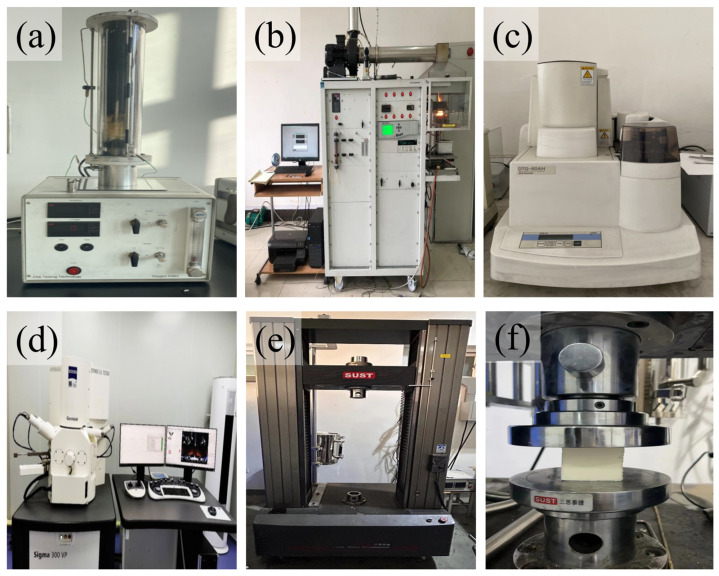
Limiting oxygen index (FTT 1402072) (**a**); Cone calorimeter (FTT 0242) (**b**), Thermogravimetric analyzer (TG/DTA DTU-2A) (**c**); Scanning electron microscopy (Sigma300) (**d**), Micro-controlled electronic universal testing machine (GTM) (**e**,**f**).

The LOI of the modified RPUF was noted using an FTT-1402072 (Fire Testing Technology Limited, East Grinstead, UK) (Figure 2a). The flame-retardant properties were tested at outflow calorimetry densities of 25, 35 and 50 kW/m^2^ using the FTT-CONE-0242 cone calorimeter (CONE, Fire Testing Technology Limited, East Grinstead, UK) (Figure 2b). Scanning electron microscopy (SEM, ZEISS Sigma300, Carl Zeiss, Berlin, Germany) (Figure 2c) was used to observe the residue morphology of RPUFs after conical combustion. The DTG-60AH (Japan Shimadzu Instruments, Kyoto, Japan) (Figure 2d) thermogravimetric analyzer was used to perform the thermogravimetric (TG) analysis of several RPUFs at a nitrogen flow rate of 50 mL/min, with a temperature range of 40~800 °C and a mass of 3~5 mg, and a heating rate of 10, 20 and 40 °C/min. According to ISO845:2006, the apparent density of the specimen was calculated using the mass–volume method, and the sample size was 50 mm × 50 mm × 50 mm. A microcomputer-controlled electronic universal (Figure 2e,f) testing machine (GTM, SUNSET, Zhuhai, China) was used to perform the compression performance test. The maximum test force of GTM microcomputer controlled electronic universal testing machine is 3000 KN, the range of force measurement was 0.1~300 KN, the test speed was 0.001~5000 mm/min and the accuracy of displacement measurement was within ±5% of the indicated value. The specimen size was 50 mm × 50 mm × 50 mm, and the setting speed was 5.0 mm/min.

## 3. Results and Discussion

### 3.1. Flammability

**Figure 3:**
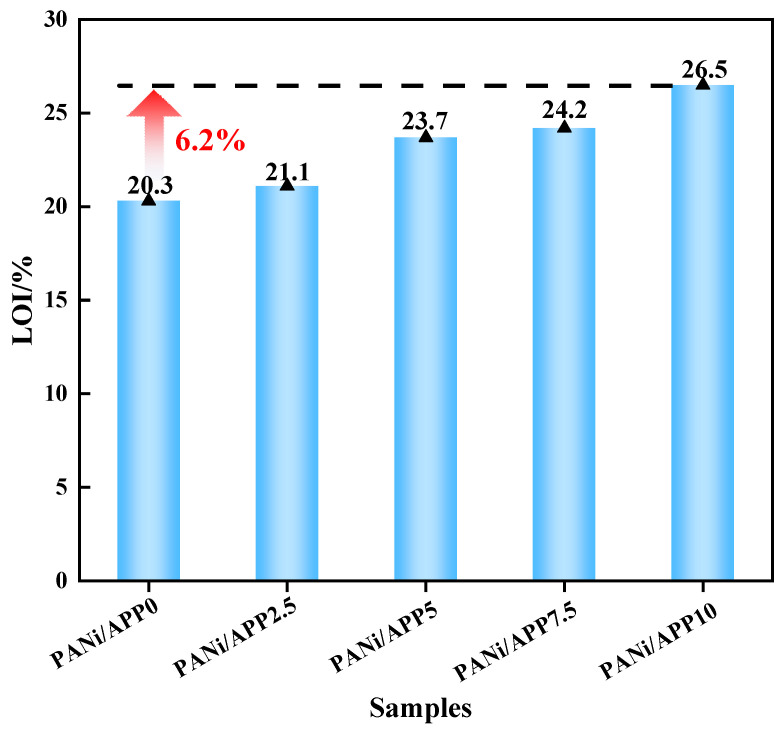
LOI of the modified RPUF.

The flammability of RPUFs was tested using LOI, and the results were shown in Figure 3. The LOI of PANi/APP0 without APP had the lowest LOI of 20.3%. However, with the increase of APP content, the LOIs of modified RPUFs were 21.1%, 23.7%, 24.2% and 26.5%. This was 0.8%, 3.4%, 3.9% and 6.2% higher than PANi/APP0, respectively. This was mainly because the polyphosphoric acid produced by APP combustion catalyzed the formation of a dense carbon layer, and the Ni phosphate salt produced by PANi acted on the carbon layer, which improved the quality of the carbon layer and inhibited the propagation of flames, thereby improving the flame retardancy of the modified material. In addition, PANi decomposed to produce active P· (PO· and PO_2_·). The gas filled in the surrounding area captured the decomposed H· and OH· of the matrix and had a quenching effect [29]. At the same time, APP combustion produced the non-combustible gases NH_3_ and H_2_O to inhibit combustion. The above results showed that PANi/APP10 had low flammability.

### 3.2. Combustibility

**Figure 4:**
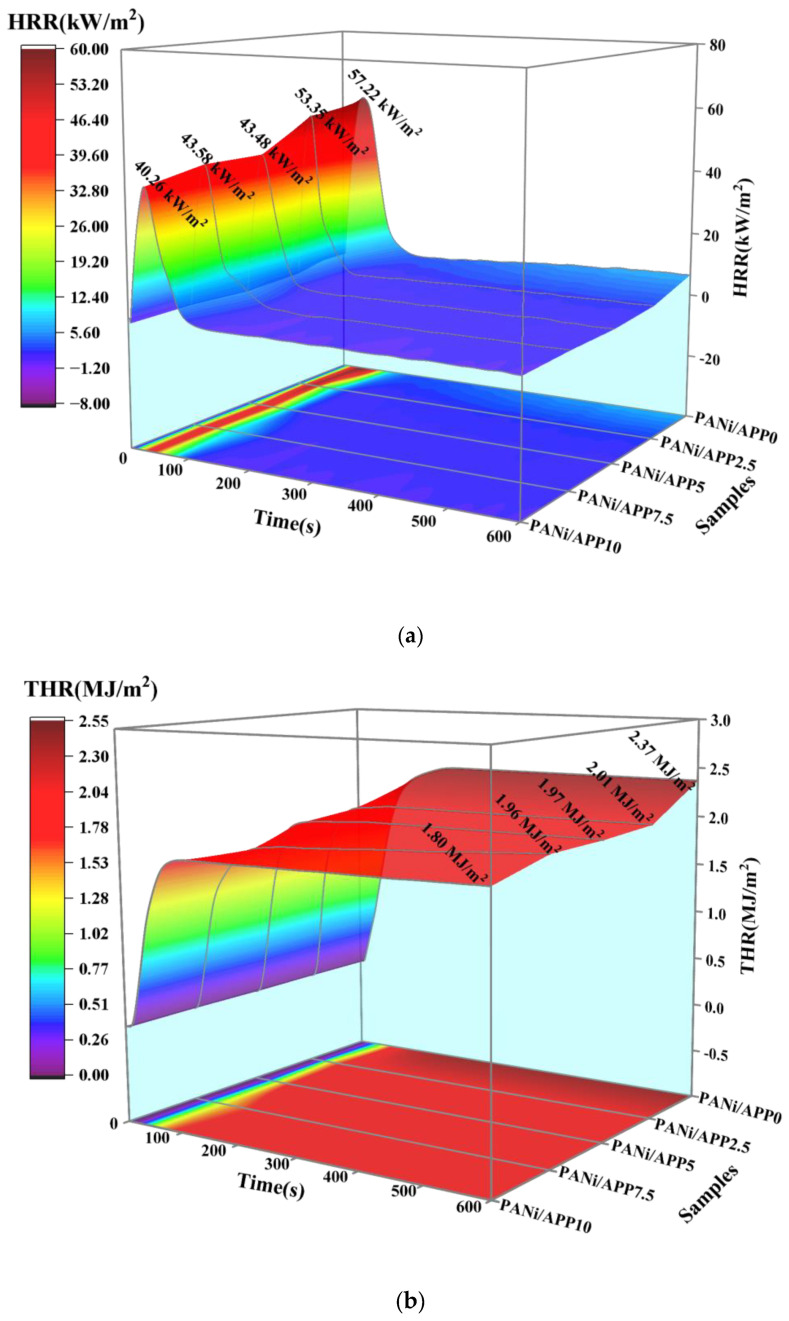

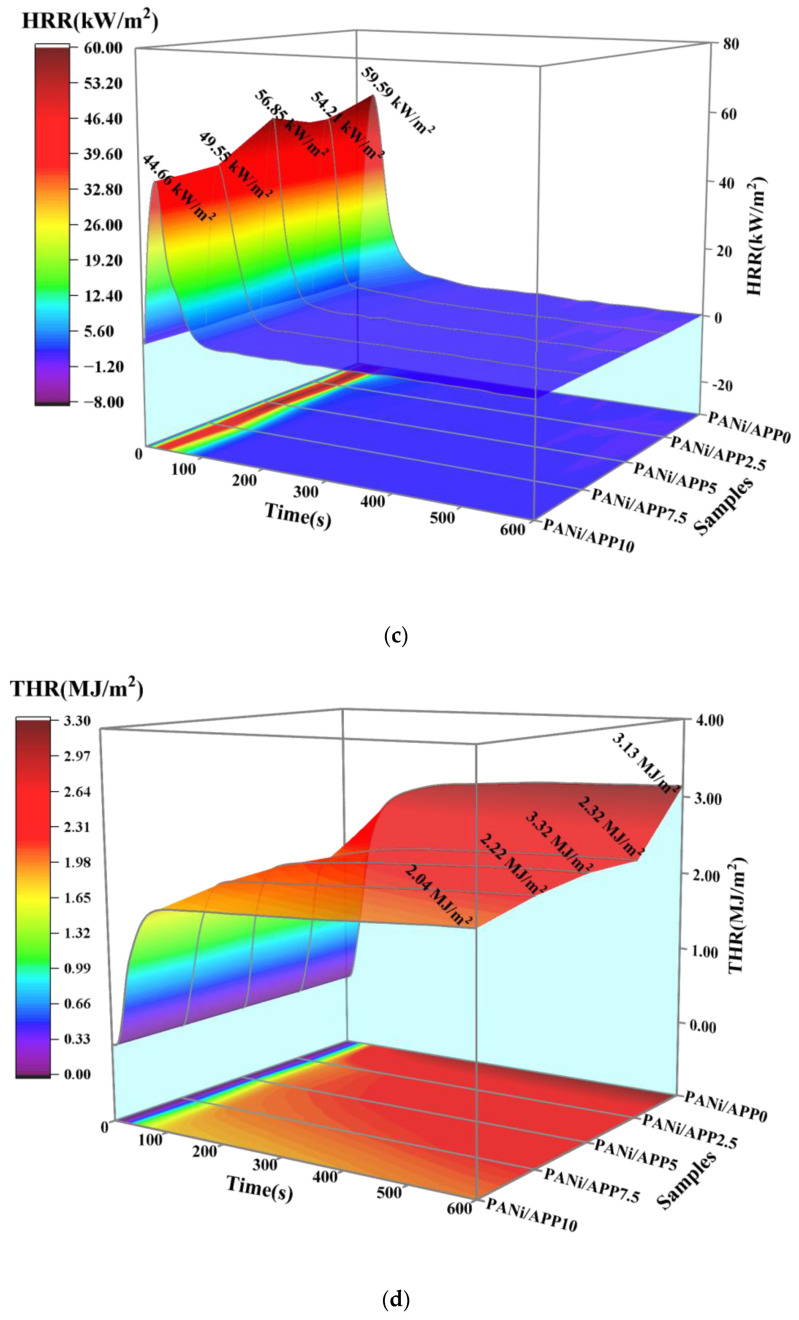

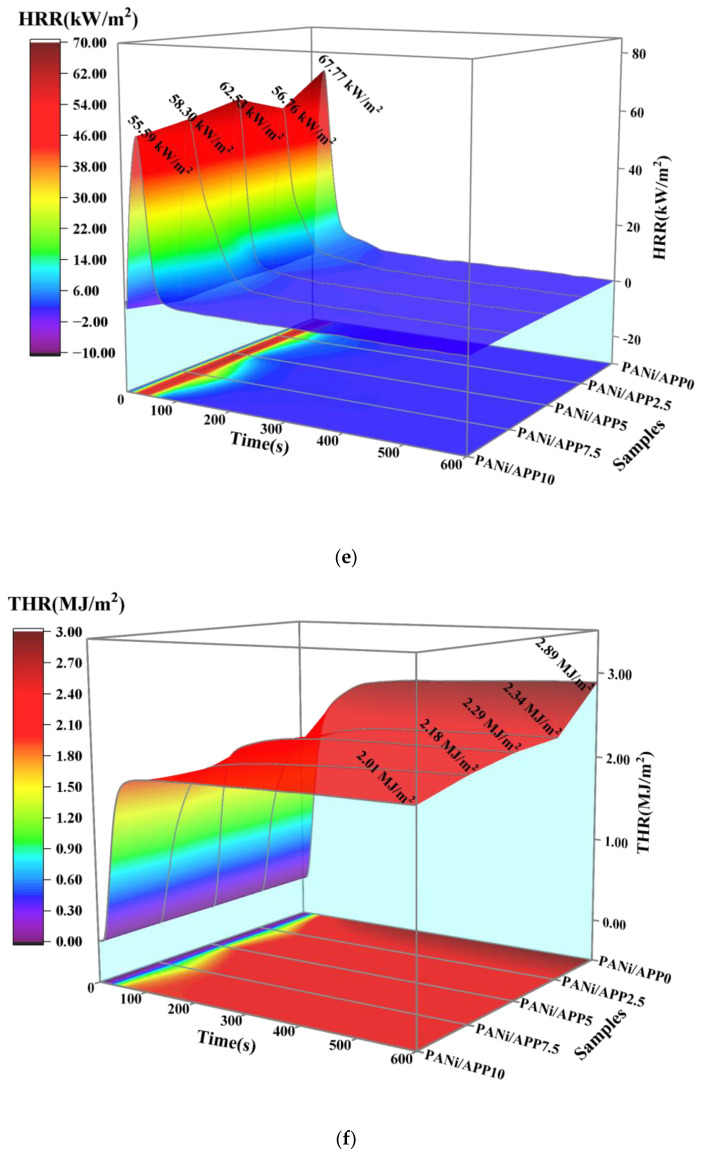
The 3D curves of HRR and THR of the modified RPUF at 25 kW/m^2^ (**a**,**b**), 35 kW/m^2^ (**c**,**d**) and 50 kW/m^2^ (**e**,**f**).

The combustion behavior of the modified RPUFs was tested using a cone calorimeter. Figure 4 shows the 3D curves of HRR and THR of the modified RPUFs at different radiation fluxes and the corresponding data. At 25 kW/m^2^, the projection of the 3D curve of the HRR showed that the exothermic process of the foam was basically completed in about the first 100 s and that there was only one exothermic peak in the combustion process. In Figure 4a,b, the PHRR and THR of PANi/APP0 were higher, at 57.22 kW/m^2^ and 2.37 MJ/m^2^, respectively, which also indicated the higher fire hazard of the unmodified foam. When APP was added to 2.5 wt%, the PHRR and THR of PANi/APP2.5 were reduced, and when the APP content was further increased, the PHRR of PANi/APP5, PANi/APP7.5 and PANi/APP10 were 43.48 kW/m^2^, 43.58 kW/m^2^ and 40.26 kW/m^2^, respectively, and the THR was 1.97 MJ/m^2^, 1.96 MJ/m^2^ and 1.80 MJ/m^2^, respectively. The PHRR and THR of PANi/APP10 were reduced to the lowest level. Compared with PANi/APP0, the PHRR and THR of PANi/APP10 were reduced by 29.64% and 24.05%, respectively. This was mainly due to the increase in APP content, and the polyphosphates produced during combustion promoted the formation of a dense, intact char layer in the modified RPUF. At the same time, the phosphorus-containing cross-linking structures (Ni–O–P and C–O–P) generated by PANi combustion acted on the char layer to improve the quality of the char layer. The more phosphorus-containing cross-linking structures that were generated, the more complete the char layer formed, and the more obvious the effect of inhibiting heat transfer. The results showed that the addition of 10 wt% APP and a PANi flame-retardant system could effectively reduce the PHRR and THR of the foams, thus improving the fire safety of the modified RPUF.

In order to investigate whether the material had the same trend under other conditions, the investigation was conducted at 35 kW/m^2^ and 50 kW/m^2^ conditions. As shown in Figure 4c,d, at 35 kW/m^2^, it can be clearly seen that PANi/APP10 had the lowest PHRR (44.66 kW/m^2^) and THR (2.04 MJ/m^2^) compared to pure PANi/APP0 with PHRR (59.59 kW/m^2^) and THR (3.13 MJ/m^2^), which were reduced by 25.05% and 34.82%., respectively. As shown in Figure 4e,f, at 50 kW/m^2^, the results also showed that PHRR (55.59 kW/m^2^) and THR (2.01 MJ/m^2^) were the lowest for PANi/APP10 compared with those of pure PANi/APP0 (67.77 kW/m^2^) and THR (2.89 MJ/m^2^), which were reduced by 17.97% and 30.45%, respectively. All three radiation conditions found that PANi/APP10 was the best flame retardant, which further supported the analysis of the LOI results.

### 3.3. Smoke Toxicity

**Figure 5:**
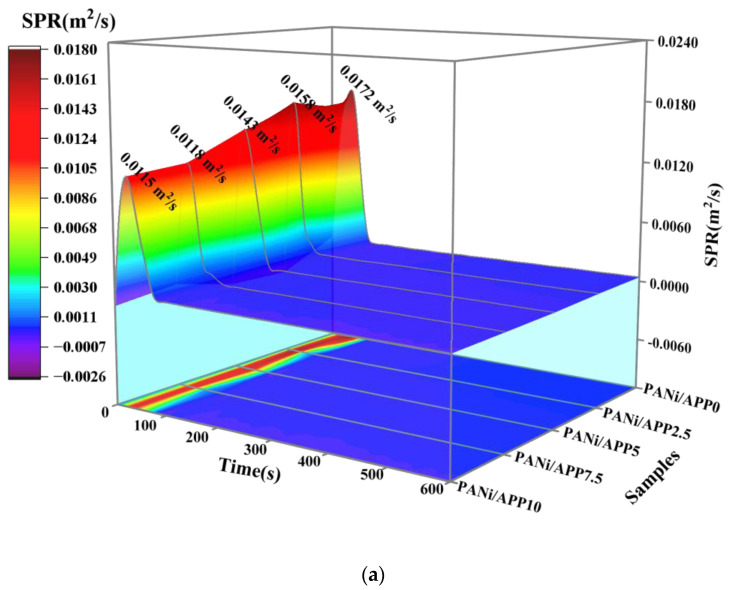

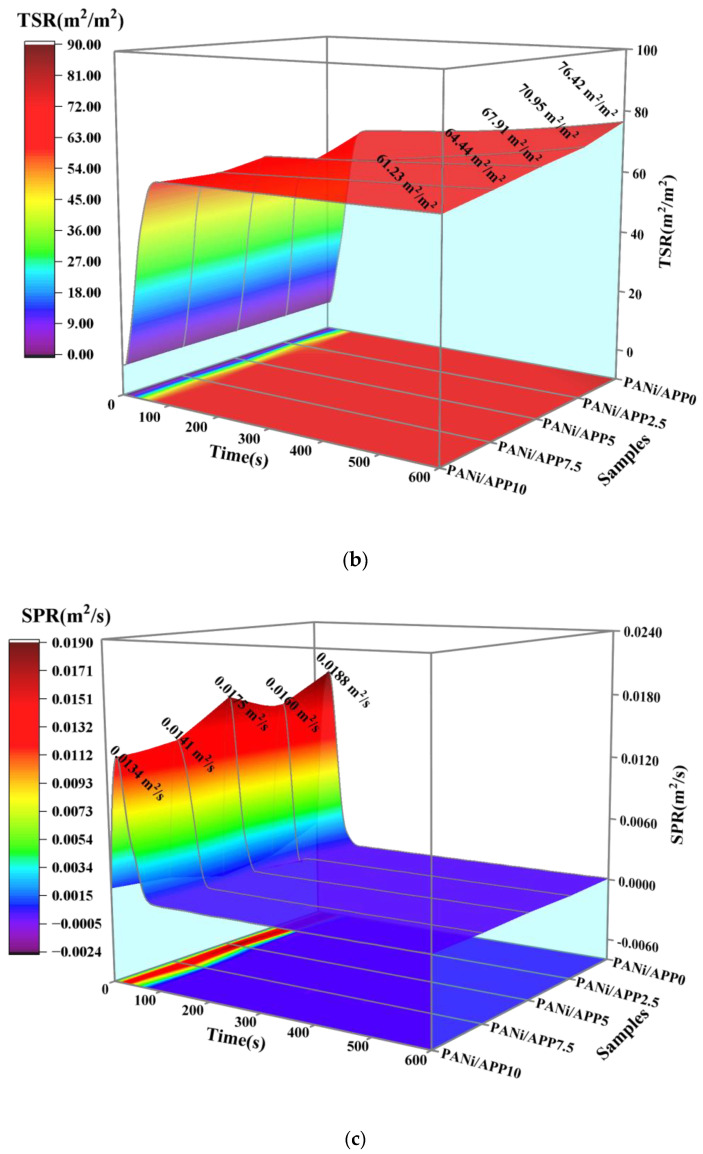

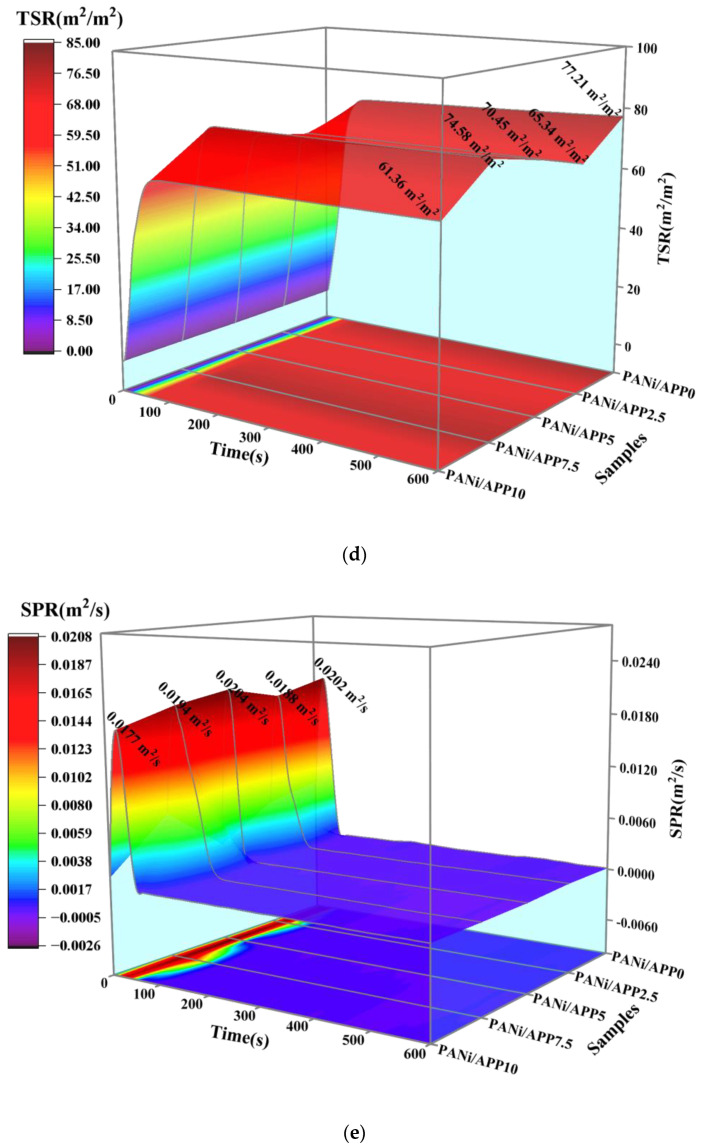

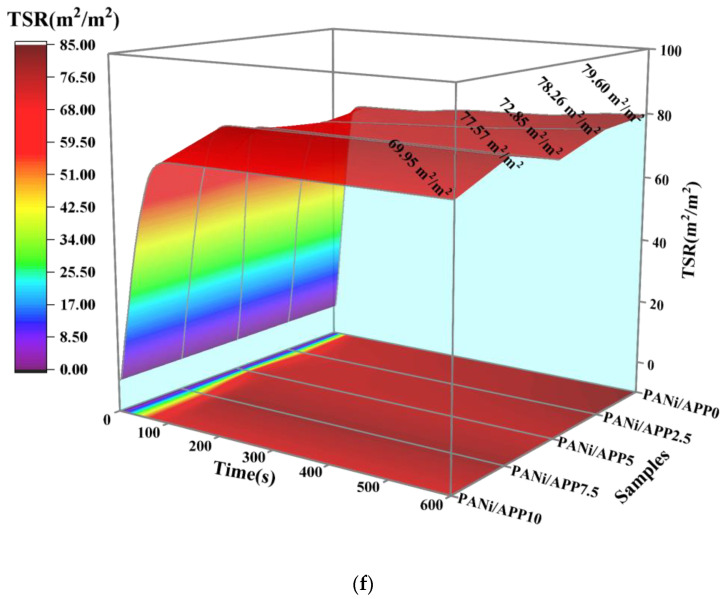
The 3D curves of SPR and TSR of the modified RPUF at 25 kW/m^2^ (**a**,**b**), 35 kW/m^2^ (**c**,**d**) and 50 kW/m^2^ (**e**,**f**).

Smoke is an important fatality factor in fires, so polymer smoke analysis is important. Figure 5 shows the 3D curves of the smoke production rate (SPR) and total smoke release (TSR) of the modified RPUF in a cone test and related data. As shown in Figure 5a,b, at 25 kW/m^2^, the SPR and TSR of PANi/APP0 were 0.0172 m^2^/s and 76.42 m^2^/m^2^, respectively. When the APP content was further increased, the SPR of PANi/APP2.5, PANi/APP5, PANi/APP7.5 and PANi/APP10 were 0.0158 m^2^/s, 0.0143 m^2^/s, 0.0118 m^2^/s and 0.0115 m^2^/s, respectively, and the TSRs were 70.95 m^2^/m^2^, 67.91 m^2^/m^2^, 64.44 m^2^/m^2^ and 61.23 m^2^/m^2^, respectively. Among them, the SPR and TSR of PANi/APP10 were significantly reduced. Compared with PANi/APP0, its SPR and THR were reduced by 33.14% and 19.88%, respectively. This was mainly because the polyphosphoric acid produced during APP combustion can increase the viscosity of the carbon layer and form a dense and intact carbon layer. At the same time, the Ni phosphate produced by PANi combustion and polyphosphate synergistically improves the shrinkage of the carbon layer, and the carbon layer was not easy to break during combustion, effectively inhibiting the release of toxic substances.

At 35 kW/m^2^ (Figure 5c,d) and 50 kW/m^2^ (Figure 5e,f), the smoke release results were basically the same. At 35 kW/m^2^, PANi/APP10 had the lowest SPR (0.0134 m^2^/s) and TSR (61.36 m^2^/m^2^), which were reduced by 28.72% and 20.53% compared with PANi/APP0. At 50 kW/m^2^, the SPR (0.0177 m^2^/s) and TSR (69.95 m^2^/m^2^) of PANi/APP10 were also the lowest, decreasing by 12.38% and 12.12% compared with PANi/APP0. This was further evidence of the analysis of the CONE results.

### 3.4. Fire Risk Assessment

Fire risk assessments are widely used to assess potential threats to polymeric materials. The most typical of these indicators are as follows: the toxic gas production index (ToxPI), the smoke production index (TSPI), the fire growth index (FGI) and the heat release index (THRI). The expressions for the four assessments had been given in detail in a previous study [30].

The ToxPI, TSPI, FGI and THRI of the modified RPUFs were shown in Table 2. It can be seen that at 25 kW/m^2^, PANi/APP10 had the lowest ToxPI (0.21 kg/s), TSPI (2.48 m^2^/s), FGI (1.25 kW/m^2^·s) and THRI (2.36 MJ/m^2^), and its fire safety was significantly higher than the other composites, which was also the same result as the cone and smoke changes. At 35 kW/m^2^, PANi/APP10 had the lowest ToxPI (0.40 kg/s), TSPI (2.51 m^2^/s), FGI (1.49 kW/m^2^·s) and THRI (2.35 MJ/m^2^). And at 50 kW/m^2^, PANi/APP10 also had the lowest ToxPI (0.53 kg/s), TSPI (2.62 m^2^/s), FGI (1.85 kW/m^2^·s), and THRI (2.38 MJ/m^2^), once again proving the best fire safety performance of PANi/APP10.

### 3.5. Carbon Layer Analysis

**Figure 6:**
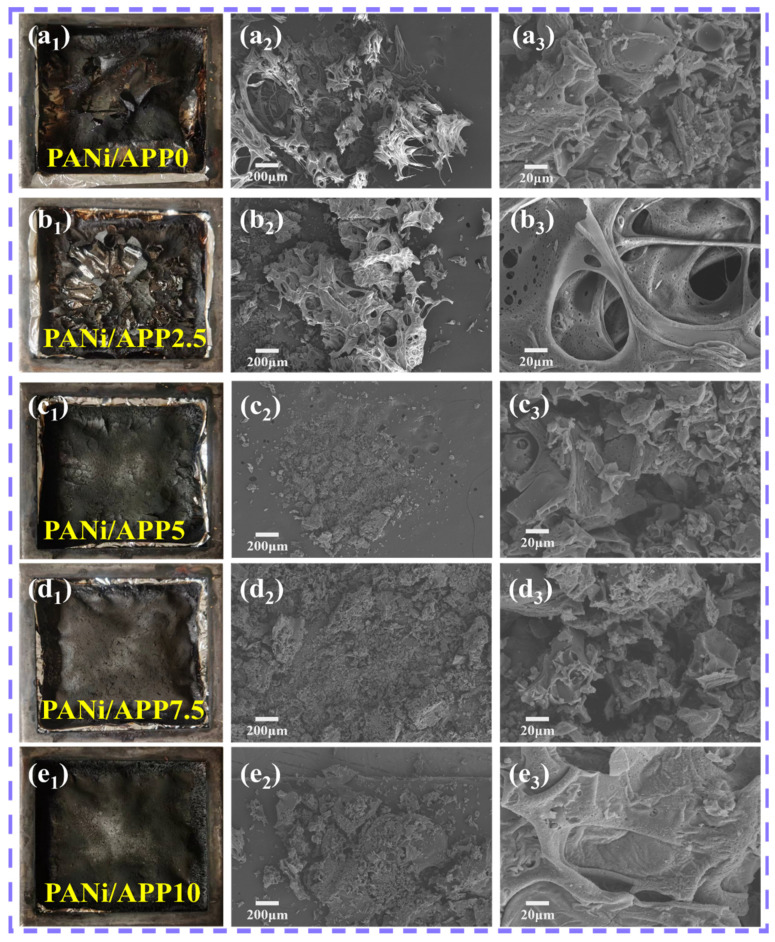
Macro photos (**a_1_**–**e_1_**) and SEM photos (**a_2_**–**e_2_**,**a_3_**–**e_3_**) of the carbon layer of the modified RPUFs after CONE combustion.

The carbon layer of the modified RPUF was further investigated by macro images and SEM images (Figure 6) after CONE testing. Figure 6(a_1_–e_1_) shows the macro images of the modified RPUFs. It can clearly be seen that the rupture area of PANi/APP0 after combustion was large, resulting in an incomplete carbon layer. The PANi/APP2.5 showed insignificant improvement in the char layer after combustion, again with more cracks, and did not inhibit the smoke release well. With the increasing APP content, it is evident that the char layers produced by PANi/APP5, PANi/APP7.5 and PANi/APP10 became smooth and dense. Among them, the char layer of PANi/APP10 was of the best quality, as well as the densest and the most complete, which can inhibit the release of smoke and toxic gases very well.

Figure 6(a_2_–e_2_,a_3_–e_3_) was the SEM image of the modified RPUFs. As can be seen from Figure 6(a_2_), there were many holes and cracks on the surface of the carbon layer of PANi/APP0. The heat was transferred through these channels to continue burning. As can be seen from Figure 6(b_2_–d_2_), the quality of the carbon layer was significantly improved, the carbon layer was thicker, and the carbon layer structure was basically well preserved. But there were still some smaller holes. In Figure 6(e_2_), the compactness of the PANi/APP10 carbon layer was significantly improved, and there were basically no holes or cracks. This was mainly due to the addition of APP, which increased the viscosity of the RPUF matrix and promoted the production of a dense carbon layer. At the same time, the polyphosphoric acid produced by APP combustion and the Ni phosphate salt produced by PANi combustion acted together on the carbon layer to improve the stability of the carbon network structure, which can maintain the integrity of the carbon layer during RPUF combustion. In turn, it effectively prevented the transfer of heat and the release of smoke. The results of the macro and SEM analysis had also explained why PANi/APP10 had a higher LOI and a lower THR and SPR.

### 3.6. Thermal Stability

**Figure 7:**
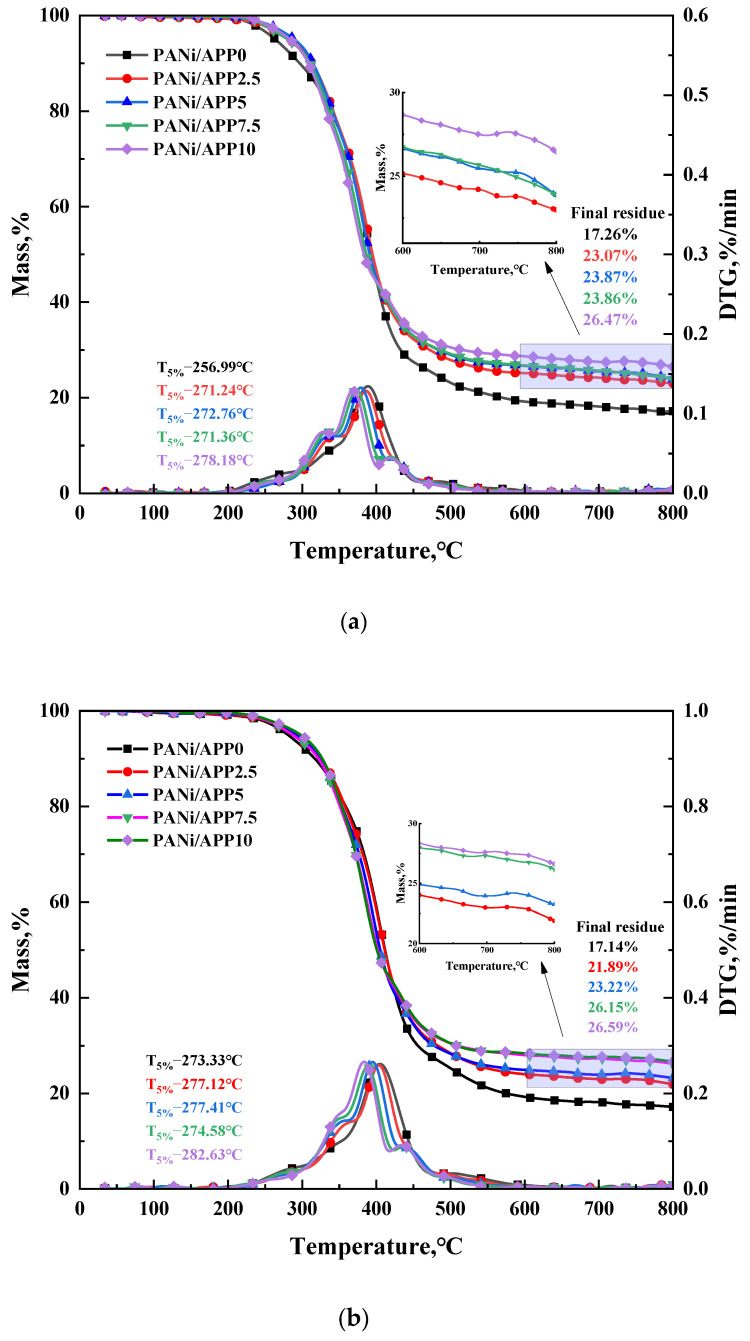

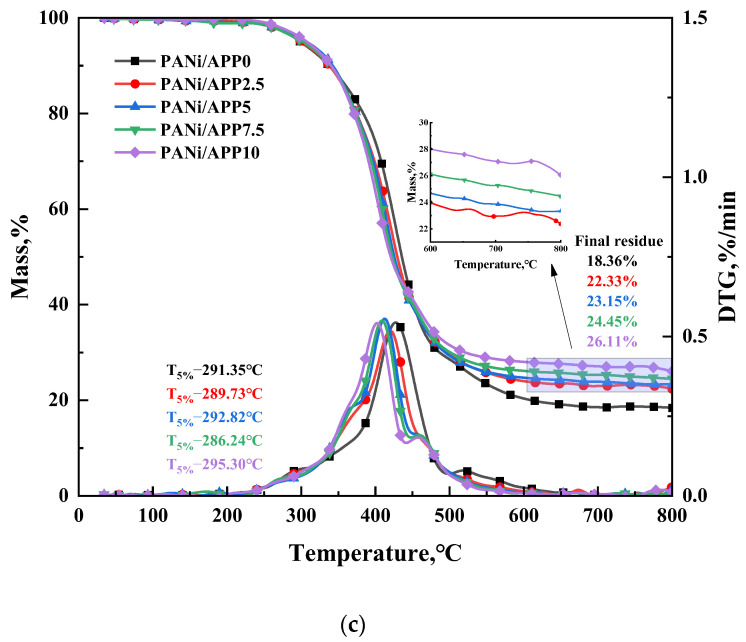
TG and DTG curves of RPUFs at 10 °C/min (**a**), 20 °C/min (**b**) and 40 °C/min (**c**) with associated parameters.

Thermogravimetric testing was performed under N_2_ conditions to investigate the thermal stability of the sample. Figure 7 shows the TG and DTG curves for each sample at different heating rates, with the associated data T_5%_ (weightlessness 5%) and carbon residue rate. Pyrolysis parameters and T_n_ (initial decomposition temperature) were listed in Table 3. As can be seen in Figure 7a, a two-step degradation process occurred for all samples. At 10 °C/min, the first decomposition stage of PANi/APP10 was 198.06–401.28 °C, and the weight loss rate was 55.86%. This stage was dominated by the hard segment decomposition of the polyurethane molecular chain, including the cleavage of chemical bonds, and the decomposition of some polyurethane bonds into polyols and isocyanates. The second decomposition stage was 401.28–800.00 °C, and the weight loss rate was 19.80%. At this stage, most of the macromolecules were destroyed by the overflow of small gas molecules such as CO. Some aromatic compounds and residues of the matrix were further decomposed and charred. And the final carbon residue rate was 26.47%.

As shown in Figure 7a, the T_5%_ of modified RPUFs were 271.24 °C, 272.76 °C, 271.36 °C and 278.18 °C. PANi/APP10 had the highest T_5%_. Compared to PANi/APP0 (256.99 °C), its T_5%_ was significantly higher. In addition, as can be seen on Table 3, the T_n_ of PANi/APP0 was 164.70 °C, and with the gradual increase of APP loading, the T_n_ of modified RPUFs was 185.68 °C, 186.77 °C, 192.08 °C and 198.06 °C. The Tn of PANi/APP10 was the highest. These results indicated that PANi/APP10 had better thermal stability. This was mainly due to the fact that the pyrophosphate produced by the thermal decomposition of APP promoted the production of a dense carbon layer by the modified RPUF, and the decomposition of Ni species in PANi formed stable Ni-O-P bonds and C-O-P bonds acting on the carbon layer, which improved the thermal stability of modified RPUFs. In addition, as shown in Figure 7a, the carbon residue rate of PANi/APP10 was 26.47% at 800 °C. It was significantly higher than that of pure PANi/APP0 (17.26%), PANi/APP2.5 (23.07%), PANi/APP5 (23.87%) and PANi/APP7.5 (23.86%). The results showed that the addition of APP had a positive effect on the charring capacity of the modified RPUF. The above results concluded that PANi/APP10 had excellent thermal stability and good charring ability.

As can be seen from Figure 7b,c and Table 3, the PANi/APP10 had the highest T_n_ and T_5%_ at 20 °C/min, at 209.46 and 282.63 °C, respectively. Compared with the PANi/APP0, they were increased by 42.57 and 9.3 °C, respectively. At the same time, the residual mass of PANi/APP10 reached 26.59%. At 40 °C/min, the PANi/APP10 also had the highest T_n_ and T_5%_, reaching 210.98 and 295.30 °C, respectively. Compared with PANi/APP0, they were increased by 25.42 and 3.95 °C, respectively. At the same time, the residual mass of PANi/APP10 reached 26.11%. The above results once again demonstrated the superiority of PANi/APP10.

The thermal stability of polymeric materials was further analyzed by evaluating the Integral programmed decomposition temperature (IPDT) [31], as a high IPDT value is an important factor for a material to have excellent thermal stability. The expression is given as:

(1) $$IPDT=AK(T_{f}\;-\;T_{i})+T_{i}$$

The IPDT of modified RPUFs under different conditions was listed in Table 3. The IPDT of PANi/APP2.5, PANi/APP5, PANi/APP7.5 and PANi/APP10 at 10 °C/min were 772.10, 791.77, 790.36 and 854.14 °C, respectively. Compared to the IPDT of PANi/APP0 (647.75 °C), it showed a significant improvement in the thermal stability of the modified RPUF with the addition of APP. The highest IPDT was obtained for PANi/APP10, which had the best thermal stability. In addition, at 20 and 40 °C/min, PANi/APP10 also had the highest IPDT of 860.83 and 851.97 °C, respectively, which were further demonstrated by the results of the TG analysis.

### 3.7. Activation Energy Analysis

Pyrolysis kinetics is equally important in determining the thermal stability of polymers. The Flynn–Wall–Ozawa (FWO) method [32], the Starink method [33], the Kissinger method [34] and the Coats–Redfern (C–R) method [35] are generally used to calculate the apparent activation energy (E) of materials. The calculation procedure of the four methods had been given in detail in a previous study [36]. Among them, the FWO method and Starink determine the stability of the polymer by calculating the average E value at different conversion rates. Kissinger’s method is used to determine the stability of the polymer by obtaining the E at T_max_. The C–R method is used to better assess the thermal stability of the material by providing the E at different stages.

Table 4 shows the E of RPUF obtained by the FWO method. The results showed that the average E of the samples after APP addition was 139.32, 132.50, 132.72 and 140.13 kJ/mol under different *α*, and the average E of PANi/APP10 was the highest. Compared with 113.30 kJ/mol of PANi/APP0, it was increased by 26.83 kJ/mol. Table 5 shows that the average E of the samples after APP additions was 136.02, 133.54, 135.57 and 138.77 kJ/mol under differences in α, and the average *E* of PANi/APP10 was the highest, which was 30.21 kJ/mol higher than that of PANi/APP0 at 108.56 kJ/mol. It can be seen that the E value of different modified foams changed significantly with changes in *α*, which was mainly caused by different heating rates. Table 6 shows the E of the modified RPUF obtained by the Kissinger method, and the results showed that the E of each foam was 122.85, 144.99, 137.95, 127.07 and 146.48 kJ/mol, respectively. It can be clearly seen that the addition of APP had a positive effect on the thermal stability of the material, and the E of PANi/APP10 was the highest, which was increased by 23.63 kJ/mol compared with PANi/APP0, which had good thermal stability. Table 7 shows the E of the modified RPUFs obtained by the C–R method. The results showed that the sum E value in the two decomposition stages of PANi/APP10 was the highest under different conditions, which was 76.87, 75.18 and 80.98 kJ/mol, respectively. Compared with PANi/APP0, the results were increased by 27.77, 22.31 and 12.54 kJ/mol, respectively. The results of the above four methods confirmed that PANi/APP10 had good thermal stability.

### 3.8. Flame-Retardant Mechanism

**Figure 8:**
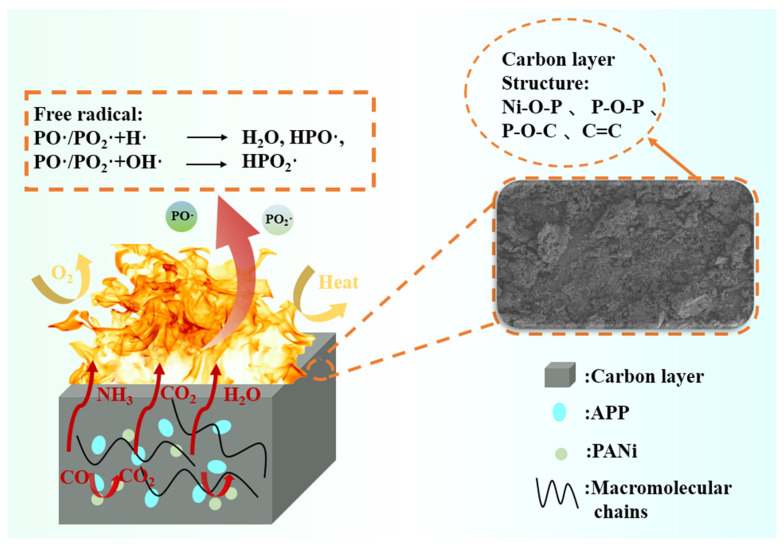
Flame-retardant mechanism of the modified RPUF.

The flame-retardant mechanism that occurs when RPUF burns was shown in Figure 8. In the gas phase, the PA molecule in PANi was heated to produce P·, which captured H· and HO·, and effectively prevented the combustion reaction. Ni ions in PANi can catalyze the formation of non-flammable gas CO_2_ from CO. At the same time, APP combustion produced non-combustible gases such as NH_3_, N_2_ and water vapor. It took away some of the heat and inhibits combustion. The produced NH_3_ can be used as a gas source to promote the charring of the modified RPUF and inhibit heat transfer. In the condensed phase, PANi combustion produced some Ni phosphate salts and some phosphorus-containing cross-linked structures (Ni–O–P and P–O–P) that adhered to the carbon layer. At the same time, APP will produce polyphosphoric acid to promote the formation of a dense carbon layer of the modified RPUF, and at high temperature polyphosphoric acid will form phosphorus-containing compounds (P–O–C) with isocyanates and attach to the carbon mesh framework. The synergistic compounds produced by the two made the carbon layer good shrinkage ability, compactness and integrity, and prevented the carbon layer from collapsing and rupturing, which reduced the release of combustibles and fumes. Therefore, the synergistic effect of the gas phase and condensed phase mechanism significantly improved the flame retardancy of the modified RPUF.

### 3.9. Mechanical Properties

**Figure 9:**
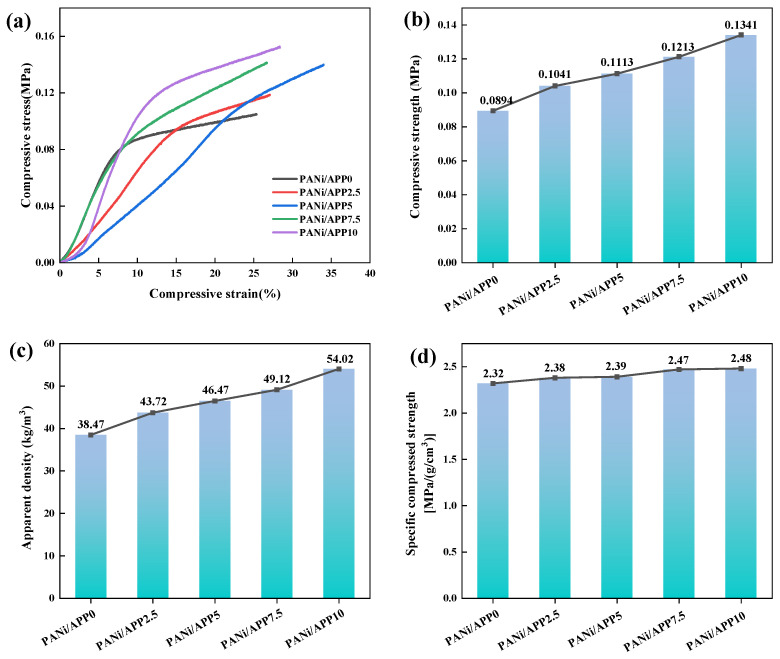
Stress–strain curves (**a**), compressive strength (**b**), apparent density (**c**) and specific compressive strength (**d**) of the modified RPUF.

Figure 9 shows the stress–strain curves and the relevant parameters of the mechanical properties of the modified foam. Figure 9a was the stress–strain curve of the modified foam and shows that the PANi/APP10 had the highest yield plateau, which also shows that PANi/APP10 had good compression performance. And Figure 9c shows the apparent density of the modified sample. It can be seen that the density of PANi/APP0 was 38.47 kg/m^3^, and the density of modified RPUF gradually increases with the increase of APP content to 43.72 kg/m^3^, 46.47 kg/m^3^, 49.12 kg/m^3^ and 54.02 kg/m^3^, respectively. The density reached its highest when the APP concentration was increased to 10 wt%. This may be due to the addition of APP, which increased the viscosity of the RPUF matrix. As can be seen in Figure 9b, with the increase of APP load, the compressive strength of PANi/APP2.5 (0.1041 MPa), PANi/APP5 (0.1113 MPa), PANi/APP7.5 (0.1213 MPa) and PANi/APP10 (0.1341 MPa) was higher than that of PANi/APP0. PANi/APP10 had the highest compressive strength. Compared with PANi/APP0 (0.0894 MPa), its compressive strength was increased by 50%, which may be related to the enhancement of APP fillers. The APP was a very fine powder that could be added to some polymers to improve flame-retardant properties while enhancing mechanical properties. In order to better evaluate the mechanical properties of the modified RPUFs, the specific compressive strength and elastic modulus were also analyzed. As can be seen from Figure 9d, with the increase of APP load, the specific compressive strength of PANi/APP2.5 (2.38 MPa/(g/cm^3^), PANi/APP5 (2.39 MPa/(g/cm^3^), PANi/APP7.5 (2.47 MPa/(g/cm^3^) and PANi/APP10 (2.48 MPa/(g/cm^3^) was higher than that of PANi/APP0. Among them, the specific compressive strength of PANi/APP10 was the largest, which was 6.90% higher than that of PANi/APP0 (2.32 MPa/(g/cm^3^). The main reasons for the improvement in mechanical properties were because the size of the APP powder grain was small and could be well compatible with PANi, and the two were not disruptive enough to destroy the cell structure of the foam when foaming. When the APP content reached 10 wt%, PANi/APP10 had the best mechanical properties.

## 4. Conclusions

APP was used as a raw material and compounded with self-made PANi to prepare a green biomass flame-retardant RPUF, and its flame retardancy, thermal stability and mechanical properties were investigated. In the LOI test, PANi/APP10 achieved the highest LOI of 26.5%. The CONE test results showed that PANi/APP10 had the lowest PHRR and THR under different conditions. Compared with PANi/APP0, the PHRR decreased by 29.64, 25.05 and 17.97%, respectively, and the THR decreased by 24.05, 34.82 and 30.45%, respectively. In terms of smoke toxicity, PANi/APP10 had the lowest SPR and TSR under different radiation intensities, while the four fire risk assessments of ToxPI, TSPI, FGI and THRI further confirmed that PANi/APP10 had lower smoke toxicity. The TG results showed that the initial temperature, IPDT and E of the modified RPUF were increased by the addition of 10 wt% APP. Therefore, PANi/APP10 had excellent flame retardancy and good thermal stability. The results of the mechanical experiments indicated that PANi/APP10 had good mechanical properties. Its apparent density, compressive strength, specific compressive strength and elastic modulus were the largest.

In addition, the experimental results showed that PANi/APP7.5 also had good performance. However, from the economic point of view, the price difference between adding 7.5% APP and 10% APP was small, and the flame-retardant effect of PANi/APP10 was obviously better than that of PANi/APP7.5, which can also be seen from the experimental results of LOI, CONE, thermal stability, smoke toxicity and compression performance. Therefore, the selection of 10% APP modified foam was the best choice in the present work. The results of this study will provide new ideas for the green flame retardant of RPUF and are of great significance for promoting the application of RPUF in aerospace and other fields.

## Figures and Tables

**Table 1:** Raw material formulation of the modified RPUFs.

Sample	3630N (g)	PM200 (g)	T9 (g)	L-580 (g)	A33 (g)	Deionized Water (g)	PANi (wt%)	APP (wt%)
PANi/APP0	40	60	0.2	1	1.8	1	3	0
PANi/APP2.5	40	60	0.2	1	1.8	1	3	2.5
PANi/APP5	40	60	0.2	1	1.8	1	3	5
PANi/APP7.5	40	60	0.2	1	1.8	1	3	7.5
PANi/APP10	40	60	0.2	1	1.8	1	3	10

**Table 2:** Fire risk assessment parameters of the modified RPUFs at different conditions.

Radiation Intensity (kW/m^2^)	Sample	ToxPI (kg/s)	TSPI (m^2^/s)	FGI (kW/m^2^·s)	THRI (MJ/m^2^)
25	PANi/APP0	0.43	2.61	1.46	2.61
	PANi/APP2.5	0.28	2.58	1.83	2.39
	PANi/APP5	0.36	2.59	1.32	2.38
	PANi/APP7.5	0.38	2.53	1.41	2.40
	PANi/APP10	0.21	2.48	1.25	2.36
35	PANi/APP0	0.52	2.58	1.86	2.53
	PANi/APP2.5	0.50	2.61	1.55	2.41
	PANi/APP5	0.57	2.53	1.96	2.41
	PANi/APP7.5	0.52	2.71	1.53	2.42
	PANi/APP10	0.40	2.51	1.49	2.35
50	PANi/APP0	0.62	2.64	2.55	2.50
	PANi/APP2.5	0.63	2.70	2.10	2.48
	PANi/APP5	0.62	2.62	2.23	2.42
	PANi/APP7.5	0.66	2.87	2.08	2.59
	PANi/APP10	0.53	2.62	1.85	2.38

**Table 3:** Pyrolysis temperature data of the modified RPUFs.

Heating Rate (°C/min)	Sample	Weight Loss Temperature Range (°C)	Percent Weightlessness (%)	Initial Decomposition Temperature (°C)	IDPT (°C)
10	RPUF-Ni/APP0	164.70–457.82	73.232	164.70	647.75
		457.82–800.00	9.39		
	RPUF-Ni/APP2.5	185.68–452.53	67.54	185.68	772.10
		452.53–800.00	8.73		
	RPUF-Ni/APP5	186.77–416.96	60.24	186.77	791.77
		416.96–800.00	15.42		
	RPUF-Ni/APP7.5	192.08–407.48	57.81	192.08	790.36
		407.48–800.00	18.19		
	RPUF-Ni/APP10	198.06–401.28	55.86	198.06	854.14
		401.28–800.00	19.80		
20	RPUF-Ni/APP0	166.89–471.32	71.13	166.89	657.84
		471.32–800.00	10.87		
	RPUF-Ni/APP2.5	203.37–477.70	68.68	203.37	752.22
		477.70–800.00	8.94		
	RPUF-Ni/APP5	205.96–431.55	59.90	205.96	779.23
		431.55–800.00	16.24		
	RPUF-Ni/APP7.5	207.17–424.26	56.51	207.17	849.07
		424.26–800.00	16.87		
	RPUF-Ni/APP10	209.46–418.06	55.38	209.46	860.83
		418.06–800.00	17.86		
40	RPUF-Ni/APP0	185.56–497.58	70.32	185.56	694.79
		497.58–800.00	10.56		
	RPUF-Ni/APP2.5	187.68–495.39	69.33	187.68	769.58
		495.39–800.00	7.952		
	RPUF-Ni/APP5	201.95–448.34	58.60	201.95	789.74
		448.34–800.00	17.20		
	RPUF-Ni/APP7.5	206.76–446.33	57.95	206.76	814.56
		446.33–800.00	16.58		
	RPUF-Ni/APP10	210.98–441.04	56.48	210.98	851.97
		441.01–800.00	17.38		

**Table 4:** The E of the modified RPUFs obtained by FWO method.

α	PANi/APP0	PANi/APP2.5	PANi/APP5	PANi/APP7.5	PANi/APP10
(%)	kJ/mol	kJ/mol	kJ/mol	kJ/mol	kJ/mol
5	95.45	139.34	121.47	156.92	148.61
10	91.83	150.49	106.68	129.63	149.57
20	100.65	135.77	123.44	115.90	112.03
30	102.48	125.35	121.76	112.87	114.42
40	112.44	133.67	133.49	122.22	122.25
50	120.52	138.49	142.36	128.90	132.67
60	122.54	143.25	144.46	134.30	131.01
70	124.18	140.60	143.04	135.96	127.86
80	125.77	143.50	143.44	143.03	156.62
90	137.10	142.73	144.83	147.46	206.24
E	113.30	139.32	132.50	132.72	140.13

**Table 5:** The E of the modified RPUFs obtained by Starink method.

α	PANi/APP0	PANi/APP2.5	PANi/APP5	PANi/APP7.5	PANi/APP10
(%)	kJ/mol	kJ/mol	kJ/mol	kJ/mol	kJ/mol
5	91.57	137.60	126.19	159.15	147.40
10	87.30	148.78	116.52	139.99	147.84
20	95.89	132.84	119.88	121.42	115.34
30	97.39	121.65	117.81	121.65	110.15
40	107.59	130.11	129.87	118.08	118.22
50	115.90	134.96	139.02	132.34	131.92
60	117.86	139.72	141.09	130.48	131.96
70	119.41	136.86	139.41	132.01	126.42
80	120.80	139.58	154.79	148.92	153.45
90	131.87	138.09	150.84	151.69	205.04
E	108.56	136.02	133.54	135.57	138.77

**Table 6:** The E of the modified RPUFs obtained by Kissinger method.

Sample	PANi/APP0 (kJ/mol)	PANi/APP2.5 (kJ/mol)	PANi/APP5 (kJ/mol)	PANi/APP7.5 (kJ/mol)	PANi/APP10 (kJ/mol)
K = −E/R	−14.78	−17.44	−16.59	−15.28	−17.62
E	122.85	144.99	137.95	127.07	146.48

**Table 7:** The E of the modified RPUFs obtained by C–R method.

Heating Rate β (°C/min)	Sample	Temperature Range (°C)	E (kJ/mol)	Heating Rate β (°C/min)
10	PANi/APP0	164.70–457.82	47.91	49.10
		457.82–800.00	1.19	
	PANi/APP2.5	185.68–452.53	58.59	66.76
		452.53–800.00	7.87	
	PANi/APP5	186.77–416.96	68.18	69.03
		416.96–800.00	0.85	
	PANi/APP7.5	198.08–407.48	64.58	68.01
		407.48–800.00	3.43	
	PANi/APP10	192.06–401.28	73.98	76.87
		401.28–800.00	2.89	
20	PANi/APP0	166.89–471.32	49.15	52.87
		471.32–800.00	3.72	
	PANi/APP2.5	203.37–477.70	51.92	61.33
		477.70–800.00	9.41	
	PANi/APP5	205.96–431.55	57.34	67.56
		431.55–800.00	10.22	
	PANi/APP7.5	197.17–424.26	57.38	61.44
		424.26–800.00	4.06	
	PANi/APP10	209.46–418.06	65.23	75.18
		418.06–800.00	9.95	
40	PANi/APP0	185.56–497.58	51.50	68.44
		497.58–800.00	16.94	
	PANi/APP2.5	187.68–495.39	50.61	67.40
		495.39–800.00	16.79	
	PANi/APP5	201.95–448.34	48.89	62.70
		448.34–800.00	13.81	
	PANi/APP7.5	206.76–446.33	49.92	65.88
		446.33–800.00	15.96	
	PANi/APP10	210.98–441.04	66.18	80.98
		441.01–800.00	14.80	

## Data Availability

Data available on request from the authors. The data that support the findings of this study are available from the corresponding author upon reasonable request.
